# Clinical efficacy and safety of Shuxuetong injection for acute myocardial infarction: A protocol for systematic review and meta-analysis

**DOI:** 10.1097/MD.0000000000024773

**Published:** 2021-02-19

**Authors:** Feng Yu, Mengxue Xin, Na Huang, Taotao Zhang, JianHui Lu, Nan Liu

**Affiliations:** a The First Affiliated Hospital of Guangzhou University of Chinese Medicine; b Guangzhou University of Chinese Medicine, Guangzhou, China.

**Keywords:** acute coronary syndrome, acute myocardial infarction, Chinese medicine, complementary therapy, meta-analysis, protocol, shuxuetong

## Abstract

**Background:**

Shuxuetong injection (SXT) is a Chinese medicine injection and has been widely used for the treatment of acute myocardial infarction (AMI) in Asia. However, whether SXT has a definite efficacy and safety is poorly understood. The aim of our study was to clarify the effect of SXT on clinical symptoms alliavation and survival in AMI patients.

**Methods:**

A systematic reviews of SXT combined with conventional therapy treating AMI will be searched in 8 electronic databases including PubMed, Cochrane Library, Embase, Wanfang Database, China Biology Medicine (CBM), Google Scholar, Chinese Scientific Journal Database (VIP), and China National Knowledge Infrastructure (CNKI), from inception to December 2020. The literature will extracted by 2 researchers independently and the methodological quality of the included study will be evaluated. We will use the Grading of Recommendations Assessment, Development and Evaluation (GRADE) system to evaluate the evidence quality of the included literature. RevMan software (version 5.3) will be applied for the original research data synthesis.

**Results:**

The results of our study will be published in a peer reviewed journal.

**Conclusion:**

Our meta-analysis will provide the latest evidence to determine whether SXT is an effective intervention for AMI patients.

## 1 Introduction

Despite advances in diagnosis and management, acute myocardial infarction (AMI) remains a life-threatening medical emergency associated with high rates of morbidity and mortality worldwide more than 1 million patients will be hospitalized for an MI or cardiac death each year in the United States alone.^[1]^ It is estimated that about 290 million patients suffer from cardiovascular diseases (CVD)in China and the total expenditure on CVD hospitalization has also increased rapidly.^[2]^ The increased burden of CVD has become the most important public health issue which should be prevented and controlled imperatively. Surgery, percutaneous coronary intervention and drugs are the 3 cornerstones of the treatment of AMI. While rates of appropriate initiation of reperfusion therapy vary widely, with up to 30% of eligible patients who are suitable for reperfusion therapy not receiving this lifesaving treatment in some registries.^[3]^ Some patients does not meet the recommended door-to-reperfusion time.^[4]^ A large proportion of patients discontinue statin use within the first year after the initial treatment and discontinuation associates with increased risk for death. Furthermore, CVD events can still occur in patients who have optimal lipid level.^[5]^ Thus, there is an urgent need for therapeutic options in this area.

As a Chinese patient medicine and has definite effects of Promoting blood circulation, reducing inflammation and detoxifification, Shuxuetong (SXT) injection has been widely used in the treatment of AMI. There are mounting studies demonstrated that SXT can improve coagulation function, inhibit the expression of inflammatory factors and alleviate ischemia symptoms.^[6–7]^

However, the role of SXT for the treatment of CVD is a matter of debate. Previous studies have been limited in their ability to provide strong evidences, such as small sample size and inconsistent adherence to modern methodological research standards, making it difficult to draw meaningful conclusions from individual trials. Therefore, a systematic review and meta-analysis to examining SXT for the management AMI is necessary.

## 2 Material and methods

### 2.1 Protocol guidance

This systematic review and meta-analysis will be conducted according to the Preferred Reporting Items for Systematic Reviews and Meta-analyses (PRISMA) guidelines.^[8]^

### 2.2 Inclusion criteria

This study evaluate the effects of SXT for patients with AMI, the diagnosis of AMI necessitates that the presence of an elevated cTn value above the 99th percentile percentile of the upper limit of the reference value, and there is an emergency clinical evidence of myocardial ischemia, such as new ischaemic ECG changes or development of pathological Q waves.^[9–10]^

#### 2.2.1 Types of participants

The target population is participants with a confirmed clinical diagnosis of AMI (as diagnosed according to clinical manifestation, ECG, cardiac enzymes) without considering any information related to their age, gender, race, education, nationality.

#### 2.2.2 Intervention methods

SXT based on conventional treatment according to relevant guideline. There is no limitation to the intervention duration and frequency. Conventional treatment is defined as percutaneous coronary intervention (PCI), thrombolytic therapy, anticoagulant and antiplatelet therapy. In addition, some symptomatic treatments, such as blood pressure control and blood lipids treatment will be included as well. At the same time, we will exclude RCT which compare SXT with other TCM injections or TCM treatment. No limitations on drug dosages or treatment courses.

#### 2.2.3 Outcomes

The primary outcome is effective rate, which depends predominantly on the alleviation in clinical symptoms and signs, ECG changes and nitroglycerin consumption. Additional outcomes include adverse drug events and numbers of deaths during the treatment and the entire follow-up period.

#### 2.2.4 Study type

Randomized controlled trials (RCTs) related to SXT for the treatment of AMI will be included, regardless of whether or not blinding method has been used. Studies should report at least the primary outcome. No language or other restrictions will be limited. Duplicate studies, those not reporting on effect estimates and providing insufficient information, or reporting on outcome measures other than the outcome of interest will be excluded. The PICOS (participants, intervention, comparison, outcome and study type) research principle is shown in the table below (Table 1)

**Table 1:** the PICOS research principle.

Participants	Intervention	comparison	Outcomes	Study type
acute myocardial infarction, follow relevant guidelines for diagnosis	SXT plus conventional therapy	conventional therapy (PCI, thrombolytic therapy, anticoagulant and antiplatelet therapy)	effective rate, adverse drug events and numbers of deaths during the follow-up period	Randomized controlled trials

### 2.3 Search strategy

The relevant articles published from inception to December 2020, regardless of language or data in 8 electronic databases: PubMed, Cochrane Library, Embase, Wanfang Database, China Biology Medicine (CBM), Google Scholar, Chinese Scientific Journal Database (VIP), and China National Knowledge Infrastructure (CNKI). We will undertake a gray literature search from ClinicalTrials.gov and the International Clinical Trials Registry Platform to identify in progress and completed trials.

We will also search manually for additional studies by cross checking the reference lists of all included primary studies and the following magazines: Chinese Journal of Evidence-Based Medicine, Chinese Journal of Integrative Medicine on Cardio-Cerebrovascuiar Disease Journal of Evidence-Based Medicine, and Chinese Journal of Evidence-Based Medicine. No language or other restrictions will be limited. The following terms will be used for the search.

1. SXT
2. SXT injection
3. 1 or 2
4. Acute myocardial infarction
5. AMI
6. myocardial infarction
7. Acute coronary syndrome
8. ST segment elevated myocardial infarction
9. Non ST segment elevated myocardial infarction
10. Or/5–9
11. RCT
12. Controlled clinical trial
13. Randomized
14. Placebo
15. Trial
16. Clinical study
17. 11 or 12–16
18. 3 and 10 and 17

### 2.4 Data collection and analysis

#### 2.4.1 Selection of studies

**Figure 1:**
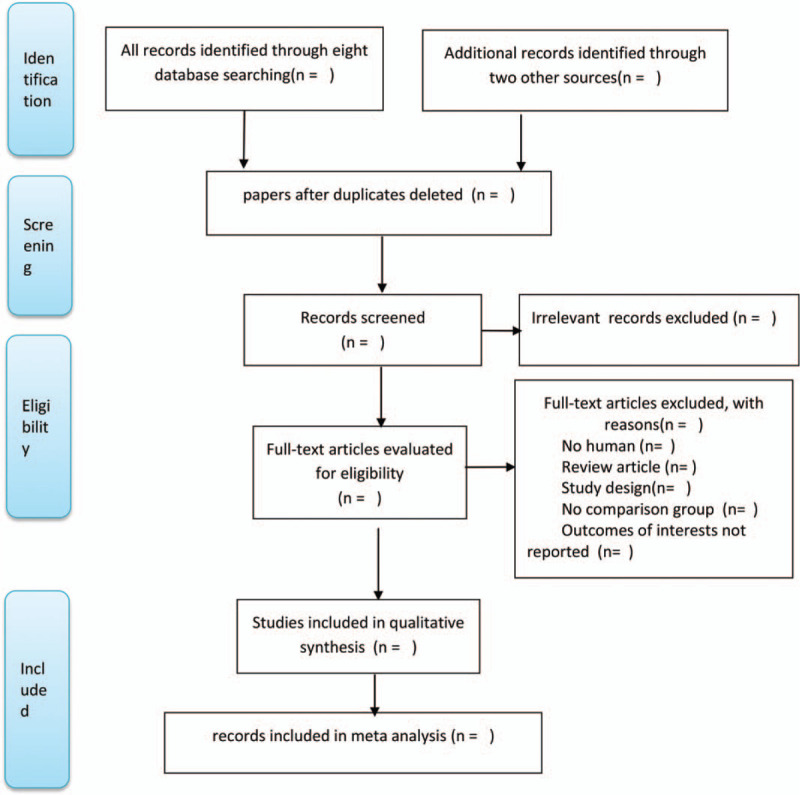
Preferred Reporting Items for Systematic review and Meta-Analysis (PRISMA) flowchart. Flow chart of study selection.

Two trained reviewers (XMX and NH) will independently screened the title and abstract to determine whether the study met the eligibility criteria. They read the full text and resolve differences through discussion. After identifying articles, they will review references from appropriate articles to identify additional references for our systematic review. The reasons for inclusion or exclusion were recorded in detail. Outcomes of interests not reported and case reports will be excluded. We will use the PRISMA flow diagram to summarize study selection processes (Fig. 1).

#### 2.4.2 Data extraction

The data extraction sheet will be prepared and applied by 2 investigators (XMX and NH) to independently extract data from each included study, including authors, publication year, study design, inclusion and exclusion criteria, age, sample size primary endpoint, follow-up duration, title, conclusion. We will contact the corresponding author for additional information if necessary. The third investigator (FY) independently reviewed the data to ensure accuracy.

#### 2.4.3 Risk of bias assessment

The methodical quality in included studies will be evaluated independently by 2 reviewers (MXX and NH) according to the risk of bias tool provided by the Cochrane Handbook for Systematic Reviews of Interventions. Seven items are included in the Cochrane Collaborations tool: random sequence generation, allocation concealment, blinding of participants and personnel, blinding of outcome assessment, incomplete outcome data, selective reporting and other sources of bias. Judgments for each item are divided into 3 levels: low risk of bias, high risk of bias and unclear risk of bias. Any disagreements will be resolved by discussion with a third reviewer (NL).

#### 2.4.4 Data analysis

The systematic reviews will be performed by using RevMan software (version 5.3) and Stata 14.0 software. The pooled odds ratios (ORs) with 95% confidence interval (95% CI) are calculated for dichotomous data (the effective rate, adverse drug events) and mean difference (MD) or standardized mean difference (SMD) with 95% confidence interval (95% CI) are calculated for continuous data (improvement of quality of life). The *I*^2^ test and the *I*^2^ statistic will be used to detect potential heterogeneity across the included studies. Heterogeneity is divided into 3 types: statistical heterogeneity, clinical heterogeneity and methodological heterogeneity, Heterogeneity was considered large if *I*^2^ > 50%. We realize that heterogeneity maybe inevitable due to the differences in length of intervention, sample size, and the drugs used. So we decide to use the random-effects model in all analysis. Begg and Egger funnel plot method will be applied to distinguish publication bias. Subgroup analyses or a meta regression will be conducted if clinical and methodological heterogeneity is found to exist. We may use narrative synthesis if meta-analysis is not appropriate.

#### 2.4.5 Subgroup analysis

Since the substantial heterogeneity or inconsistency may exist among included studies, we will perform subgroup analysis for different intervention forms to explore whether the effects are different in subgroups, we will take study quality, the degree of AMI severity, the age, geographical area and other different control interventions into consideration. We will also calculate the incidence rates of different types of adverse events if enough research is available.

#### 2.4.6 Sensitivity analysis

Sensitivity analysis will be applied to assess the robustness and stability of pooled outcomes after removing the inferior quality papers. The results will depend on the missing data, risk of bias, sample size, and quality of methods of each study.

#### 2.4.7 Assessment of publication biases

Stata 14.0 (Stata Corp, College Station, TX) will be conducted for statistical investigation and a funnel plot analysis will be drawn to assess the publication bias if there are more than 10 studies included.

#### 2.4.8 Assessment of quality of evidence

We will use the Grading of Recommendations Assessment, Development, and Evaluation (GRADE) to evaluate the results. In the GRADE system, the quality of evidence will be categorized into 4 levels: high, moderate, low, and very low quality.

#### 2.4.9 Ethics and dissemination

The study was designed and led by an executive steering committee, and the ethics approval was passed by the ethics committee of the first affiliated hospital of Guangzhou university of Chinese Medicine. Our research results will be disseminated in a peer reviewed publication. If adjustments are needed, we will update our protocol to include any changes in the whole process of research.

## 3 Discussion

Coronary artery disease is the most common cause of death and its frequency is increasing worldwide. STEMI is relatively more common in younger than in older people, and more common in men than in women.^[11]^ We have succeeded in creating a cohort of survivors of AMI complications who live longer, but experience considerable morbidity and poor quality of life. Therefore, it is urgent to explore effective methods for acute myocardial infarction. According to the theory of Chinese medicine, “Phlegm and blood stasis stagnation” is the core pathogenesis of AMI, reducing phlegm and removing blood stasis is an important principle for the management of AMI.^[12]^ As a representative medicine of promoting blood circulation, SXT injection has been used in clinical practice for many years with definite clinical efficacy. However, no single trial has been adequately powered to test for such cardiovascular efficacy, there is no systematic review to evaluate the efficacy of SXT for the treatment of AMI and no recommendation for Chinese medicine intervention in the latest treatment guidelines.^[10]^ Contemporary prevention and treatment of AMI patients has entered an “evidence-based coronary care phase,” driven by relevant guidelines. We have searched 3 major registration sites, PROSPERO international systematic review platform (https://www.crd.york.ac.uk/PROSPERO/), Open Science Framework (OSF, https://osf.io/) and Cochrane network (https://www.cochrane.org/), there is no registered systematic review related SXT for AMI. Therefore, we conduct this study to summarize and analyze the current evidence regarding whether SXT is efficacious at reducing the fatality rate in the treatment of AMI, to gain more reliable evidence for the use of Chinese Medicine in cardiovascular disease.

## Author contributions

**Conceptualization**: Feng Yu.

**Data curation**: Feng Yu, Mengxue Xin, and Na Huang.

**Formal analysis**: Feng Yu, Mengxue Xin.

**Methodology**: Na Huang, Nan Liu.

**Software:** Mengxue Xin, Na Huang.

**Supervision:** Na Huang, Jianhui Lu and Nan Liu

**Visualization:** Taotao Zhang and Jianhui Lu.

**Writing – original draft:** Mengxue Xin, Na Huang and Jianhui Lu.

**Writing – review & editing:** Feng Yu, Mengxue Xin.
